# Mapping bound plasmon propagation on a nanoscale stripe waveguide using quantum dots: influence of spacer layer thickness

**DOI:** 10.3762/bjnano.6.208

**Published:** 2015-10-19

**Authors:** Chamanei S Perera, Alison M Funston, Han-Hao Cheng, Kristy C Vernon

**Affiliations:** 1 Queensland University of Technology, Brisbane 4001, QLD, Australia; 2 School of Chemistry, Monash University, Clayton 3800, VIC, Australia; 3 Australian National Fabrication Facility-QLD Node, AIBN, University of Queensland, St. Lucia 4072, QLD, Australia

**Keywords:** photoluminescence, plasmonics, quantum dot, spacer layer, stripe waveguide

## Abstract

In this paper we image the highly confined long range plasmons of a nanoscale metal stripe waveguide using quantum emitters. Plasmons were excited using a highly focused 633 nm laser beam and a specially designed grating structure to provide stronger incoupling to the desired mode. A homogeneous thin layer of quantum dots was used to image the near field intensity of the propagating plasmons on the waveguide. We observed that the photoluminescence is quenched when the QD to metal surface distance is less than 10 nm. The optimised spacer layer thickness for the stripe waveguides was found to be around 20 nm. Authors believe that the findings of this paper prove beneficial for the development of plasmonic devices utilising stripe waveguides.

## Introduction

Plasmons are a coherent oscillation of electrons in a metal [1]. Loosely bound electrons can combine with incoming photons and propagate along the metal/dielectric interface. These charge density waves create a strong near-field [1]. There is increasing demand for high speed data communication as well as miniaturised devices, and plasmonics is a possible solution that can provide both the high speed and miniaturisation [2–3]. Plasmonics enables the squeezing of optical waves into miniscule structures and manipulating these waves to achieve all-optical circuits. Metal waveguides are a popular method to route light in nanoscale all-optical circuitry. Of all the waveguides, stripe waveguides are popular due to their ease of fabrication as well as the ability to support plasmon modes having relatively high propagation lengths [4–5]. These special modes are called long range surface plasmon polaritons (LRSPPs). LRSPPs have been shown to have large propagation lengths in the visible light range, greater than 10 µm [6].

When quantum dots (QDs) are placed in the vicinity of propagating plasmons, QDs can be locally excited by the plasmon if the plasmon frequency lies within the absorption spectral range of the QDs [1,7]. Photoluminescence of the QD occurs due to excitation of the QD by the incident field of the propagating SPP on the stripe.

Intensity of the QD photoluminescence (PL) arising from the propagating plasmons is proportional to the intensity of the local electric field at the given position [7–8]. Therefore, QDs can be used to map the propagating plasmons on a waveguide [9–10]. It is well known that QD PL can be quenched via non-radiative transition of energy from QD to the metal if the QDs are placed too close to the metal surface [7,11]. Therefore, optimisation of the distance between the QD and metal surface is vital to enhance the PL intensity.

In this paper we present the mapping of the above bound plasmon mode using quantum dot photoluminescence. For a plasmonic stripe waveguide, we demonstrate that QD to waveguide surface distance is a critical factor on the QD PL [11]. We use degree of polarisation (DoP) measurements to prove that QD PL in the vicinity of the metal waveguide is arising from the local excitation of QDs by the propagating plasmon. Our experimental findings are supported by finite element modelling using COMSOL Multiphysics. Authors believe that findings of this work will prove beneficial in studying light matter interaction in nanoscale devices and all-optical circuitry.

## Theory

**Figure 1:**
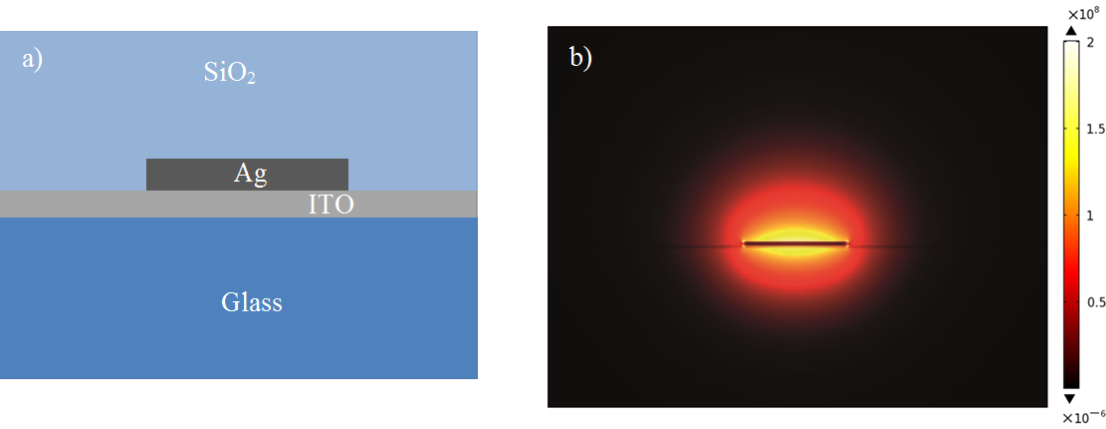
(a) The schematic diagram of the stripe waveguide covered with SiO_2_ on an ITO-coated glass substrate, and (b) The magnitude of the electric field of the bound LRSPP mode supported by a 750 nm wide, 30 nm thick silver stripe waveguide on an ITO coated glass substrate at an excitation wavelength of 633 nm.

COMSOL Multiphysics was used to run simulations on silver stripe waveguides supported on an indium tin oxide (ITO)-coated glass substrate with poly(methyl methacrylate) (PMMA) cladding. The width of the waveguide was chosen to be 750 nm to visualise easily under an optical microscope. Silver stripe waveguides were designed to be excited using a 633 nm laser. Permittivities of the materials used in modelling were, Silver as −16.4 + 1.13*i* [12], ITO as 3.42 + 0.22*i* from Sopra database, glass 2.3, and SiO_2_ as 2.4 [13]. The thickness of the ITO was 15 nm. Geometrical parameters of the stripe waveguide were theoretically determined to ensure the waveguide was single mode (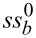). This required the thickness of the waveguide to be around 30 nm. The propagation length of this mode within a waveguide of these parameters has been previously reported experimentally to be 18 ± 6 µm [6]. The electric field profile of this mode is shown in Figure 1 below.

The grating periodicity (*a*) was calculated to provide stronger incoupling to the desired mode using [14]:

[1]
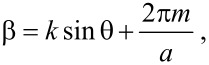


where β is the wavenumber of the plasmon mode, *k* is the wavenumber of the incident light, θ is the angle of incidence and *m* is the order of the grating. For a 1st order grating at normal incidence of light θ = 0 and *m* = 1. The calculated grating periodicity was 416 nm. The groove-to-pitch ratio was chosen as 1/2 [7].

## Experimental

**Figure 2:**
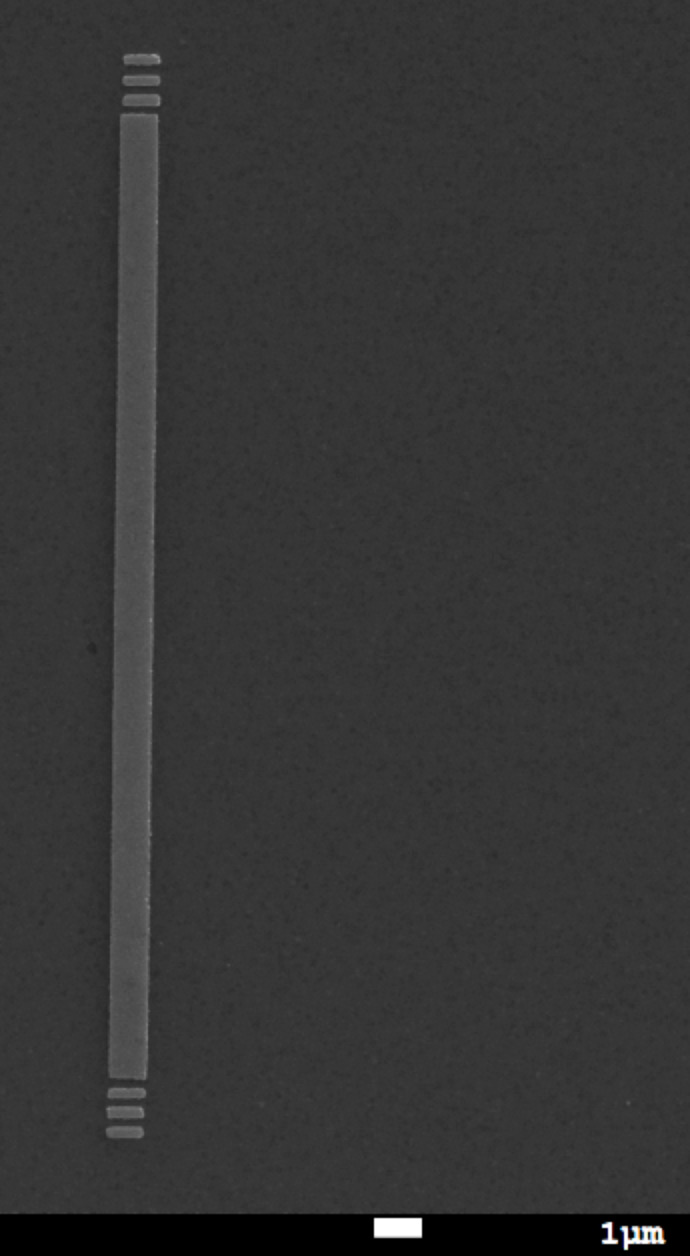
SEM image of 20 µm stripe with grating periodicity of 408 nm.

We fabricated stripes 30 nm thick, 750 nm wide with grating using electron beam lithography (EBL). Bilayer PMMA (950k A4 and 495k A4 PMMA from Microchem GmbH) was patterned using EBL and then developed for 30 seconds in MIBK:IPA developer solution. A silver film with 30 nm thickness was evaporated onto the resist using PVD 75 electron beam evaporator under 0.1 Å/s. Lift-off of the resist was achieved in an acetone bath. Stripes with length 20 µm were fabricated (Figure 2). When using bilayer PMMA in EBL, the substrate must be relatively conductive. We used ITO-coated glass substrate as a conductive substrate. The presence of the ITO layer affects the plasmonic mode propagation, and thus the ITO layer is included in the FEM simulation. The ITO layer affects the propagation length and loss of the wavenumber of the guided mode and any cut-off thicknesses for particular modes. For more information see [6].

**Figure 3:**
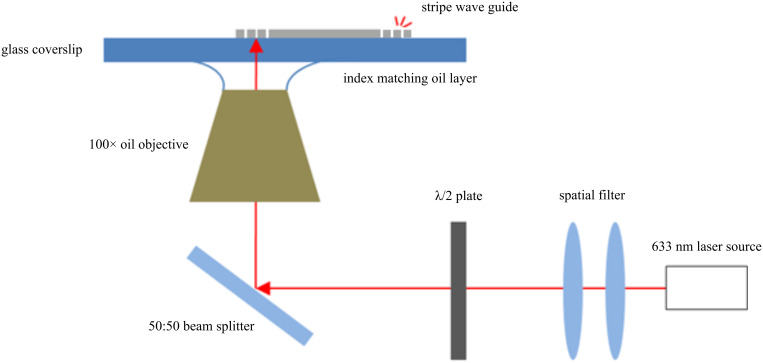
Conceptual representation of the excitation setup. The stripe waveguide is excited using the input gratings and light outcoupling is observed at the opposite end.

Fabricated structures were then optically tested for plasmon propagation. The sample was mounted on an inverted microscope stage. A laser beam was focused onto the input grating through the high numerical aperture (NA 1.3) 100× oil objective in contact with the backside of the sample through index matching oil (Figure 3). The outcoupled light from the opposite end of the waveguide was observed. CCD image of the outcoupling at the stripe end is shown in Figure 6b.

**Figure 4:**
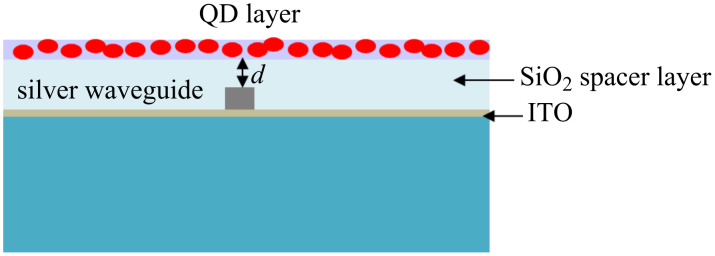
Schematic diagram of the cross section of the sample. Silver stripe waveguide on ITO-coated glass slide. Structures are covered with SiO_2_ spacer layer with thickness *d*. QDs were spin coated on top of SiO_2_.

Finally, the samples were covered with SiO_2_ spacer layers of thicknesses 5, 10, 15, 20, 25, 30, 40, and 50 nm using a PVD 75 electron beam evaporator. Quantum dots with an emission wavelength of 655 nm were obtained from invitrogen (Cat. No: Q21321MP). These carboxyl QDs are made from CdSe nanocrystals shelled with a ZnS layer. The core–shell material is further coated with a polymer layer to allow for better dispersion of the QDs in aqueous solution. These QDs have a narrow symmetric emission band with a maximum at 655 nm and are about 10 nm in size. Above QDs were diluted 50× in deionised water and spin coated onto the SiO_2_ surface. Under these conditions the QD layer is highly dense. A schematic diagram of the sample cross-section is depicted in Figure 4 (image is not to scale).

The above experimental arrangement was modified to include a filter setup (632.8 nm excitor/649 nm single-edge dichroic beam splitter/655 nm single band bandpass filter) in the microscope for the QD emission line of 655 nm. Plasmon excited QD PL was observed at 655 nm following excitation of the stripe waveguide at 633 nm. Incident intensity of the laser was 2.52 mW/mm^2^.

## Results and Discussion

**Figure 5:**
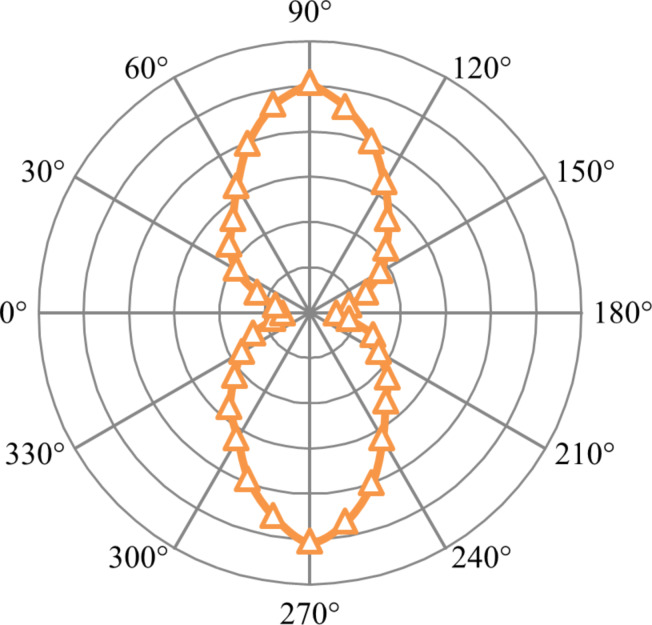
Polar plot for plasmons on 20 µm long stripe waveguide excited via grating coupling. Radial scale goes from 0 to 1.

The polarisation dependence of the outcoupled light from the stripe waveguides (LRSPP mode) in the absence of a QD layer was investigated by varying the polarisation of the incoming excitation light (refer Figure 5). For each angle outcoupling intensity in the captured CCD image was analysed. Measured intensity values were normalised w.r.t. the incident laser intensity at the waveguide input.

Degree of Polarisation, DoP = (*I*_max_ – *I*_min_)/(*I*_max_ + *I*_min_), analysis [10] shows that the outcoupled light is 78.9% TM polarised, which is in good agreement with other works and indicates the presence of plasmons [10]. This verifies that the far-field outcoupling observed at the end of the waveguides is a direct result of the propagating plasmons on the stripe.

**Figure 6:**
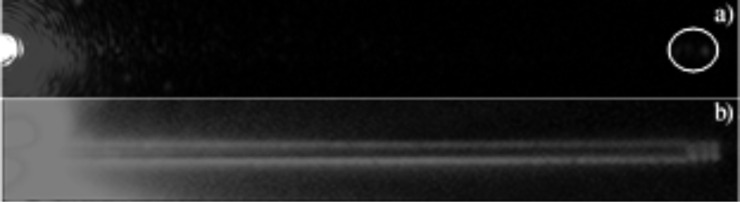
20 µm stripes a) plasmon outcoupling when excited via grating coupling, b) QD coupling. Spacer layer 20 nm.

CCD images of the plasmon outcoupling without QD and with QD are shown in Figure 6. When the LRSPP mode on the stripe waveguide was excited, the excited plasmon propagated along the stripe and scattered into the far field at the end of the waveguide or at a surface defect as shown in Figure 6a. When the waveguides were coated with a homogeneous QD layer (with a spacer layer of thickness 20 nm), we observed the bright luminescence around the waveguide rapidly decaying along the waveguide length (Figure 6b). If the frequency of the plasmons propagating on the stripe lies within the broad absorption band of the QDs, QDs in the immediate vicinity of the waveguide can be excited via propagating plasmons resulting in PL around the waveguide. We interpret the brighter QD PL along the waveguide edges is arising due to scattering of the propagating plasmons due to surface defects present along the edges. Intensity of the QD PL should be propotional to the intensity of the local field at the position of the QD. Therefore, the QDs placed in the evanescent tail of propagating plasmons provide a convenient method to probe plasmon propagation.

**Figure 7:**
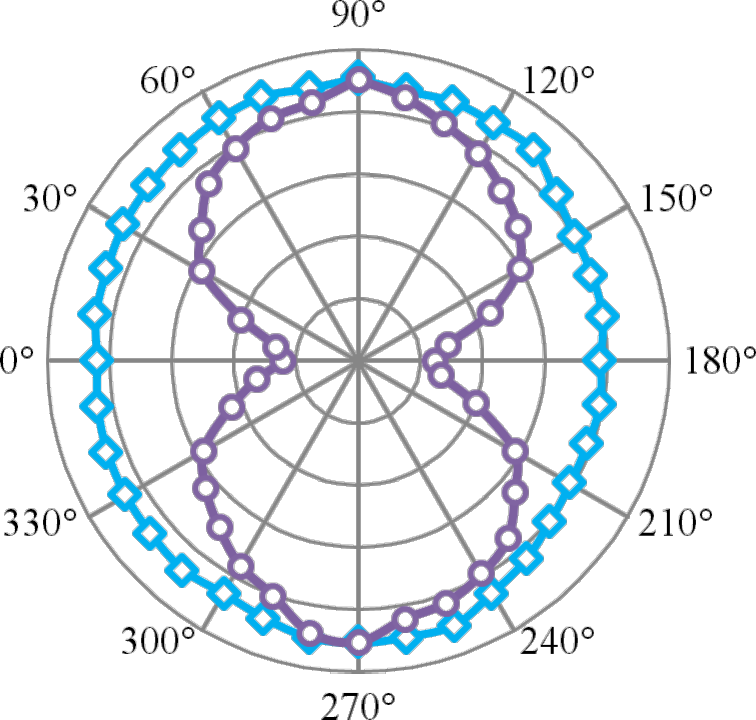
Polar plot for plasmon excited (circles) and b) bulk QDs (squares). The radial scale goes from 0 to 1.

Photoluminescence from the QDs where there with no waveguides in the vicinity (bulk QDs) was analysed for polarisation dependancy of the excitation laser (refer Figure 7, curve represented by the squares). DoP analysis showed that bulk QDs show a very little degree of polarisation (~8% TM polarised). These QDs are slightly elongated in the direction of its crystal axis. The light emission of these QDs is preferentially polarised along the crystal axis [15]. The random orientation of this crystal axis in QDs in the film may result in nearly unpolarised behaviour in bulk QDs [16–17]. Then the stripe waveguides were excited using 633 nm laser light. The polarisation of this laser beam was varied and the QD PL near the waveguide surface was observed (refer Figure 7, curve represented by the circles).

As observed earlier, plasmons on the supported waveguides were 78.9% TM polarised. DoP analysis showed that the QD PL near the stripe waveguide showed 57.5% TM polarisation. In simple terms, the QD PL has an increased PL intensity when excited using a TM polarised laser. This increase in the DoP is an evidence that plasmons are causing the near-waveguide luminescence and QDs near the waveguide are excited by the propagating surface plasmons on the waveguide.

**Figure 8:**
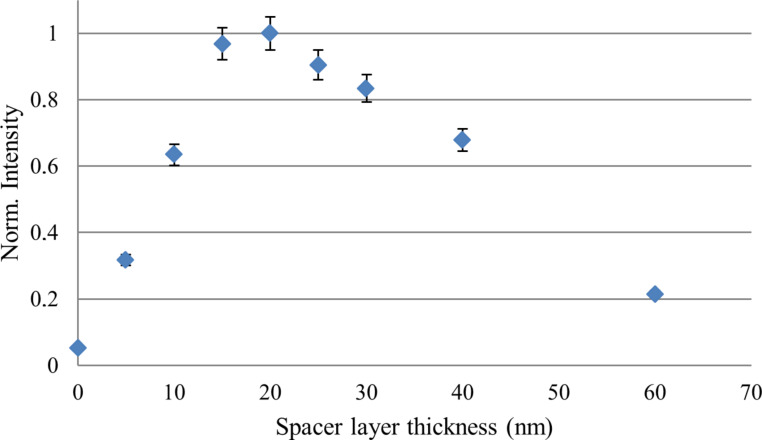
Normalised intensity at the outcoupling end of 20 µm stripe against spacer layer thickness.

PL of the QDs can be quenched due to non-radiative decay into ohmic losses in the metal if placed too close to the metal surface of the waveguide [11]. This can be eliminated by inserting a dielectric spacer layer between QDs and the waveguide. In this experiment we used SiO_2_ as the dielectric spacer layer. The SiO_2_ layer acts as a spacer layer as well as helps to keep the excited LRSPP mode bound. The PL of QDs at the 20 µm long stripe end was measured as a function of spacer layer thickness (Figure 8). Intensities are normalised with respect to the incident light intensity. For each spacer layer, outcoupling intensity from five different stripes were obtained and averaged values are used in the graph below.

We observed that a spacer layer with a thickness of ca. 20 nm gives the highest outcoupling intensity. This is expected from theoretical calculations of single QD coupling to stripe waveguides [11]. When the QD to waveguide surface distance is less than 10 nm, non-radiative decay into metallic losses is prominent. Hence, the outcoupling intensity at the end is decreased and our observation is consistent with previously reported values [10,18]. After 30 nm, the distance between QD and waveguide surface increases and the QD is positioned far from the evanescent surface plasmon mode tail. Therefore, QD coupling in to plasmon-mediated free charge carriers is lower at larger distances.

## Conclusion

We demonstrated that the QD PL can be used to image LRSPP propagating on a stripe waveguide. The optimised PL can be obtained at a spacer layer thickness of approx. 18 nm. QD photoluminescence is quenched due to ohmic losses when the spacer layer thickness is less than 10 nm. Polar plots proved that the QDs in proximity to the waveguide surface were excited by the evanescent filed of the propagating plasmons.
